# Anatomy of a Neotropical insect radiation

**DOI:** 10.1186/s12862-018-1146-9

**Published:** 2018-03-14

**Authors:** Isaac Winkler, Sonja J. Scheffer, Matthew L. Lewis, Kristina J. Ottens, Andrew P. Rasmussen, Géssica A. Gomes-Costa, Luz Maria Huerto Santillan, Marty A. Condon, Andrew A. Forbes

**Affiliations:** 1 Department of Biology, Cornell College, Mount Vernon, Iowa, 52314 USA; 2 Systematic Entomology Laboratory, U.S. Department of Agriculture, Beltsville, MD 20705 USA; 3 Department of Biology, University of Iowa, Iowa City, Iowa 52242 USA; 4 Programa de Pós-Graduação em Biologia Vegetal, Universidade Federal de Pernambuco, Av. Prof°. Moraes Rego s/n, Cidade Universitária, Recife, Pernambuco 50670-901 Brazil; 5 Departamento de Entomología Museo de Historia Natural, Universidad Nacional Mayor de San Marcos, Av. Arenales 1256, Apartado 14-0434, 14 Lima, Peru

**Keywords:** Speciation, Lineage through time (LTT) plots, Neutral theory, Diversification, Host-use, Tropics, Herbivorous insects, Parasitoids

## Abstract

**Background:**

Much evolutionary theory predicts that diversity arises via both adaptive radiation (diversification driven by selection against niche-overlap within communities) and divergence of geographically isolated populations. We focus on tropical fruit flies (*Blepharoneura*, Tephritidae) that reveal unexpected patterns of niche-overlap within local communities. Throughout the Neotropics, multiple sympatric non-interbreeding populations often share the same highly specialized patterns of host use (e.g.*,* flies are specialists on flowers of a single gender of a single species of host plants). Lineage through time (LTT) plots can help distinguish patterns of diversification consistent with ecologically limited adaptive radiation from those predicted by ecologically neutral theories. Here, we use a time-calibrated phylogeny of *Blepharoneura* to test the hypothesis that patterns of *Blepharoneura* diversification are consistent with an “ecologically neutral” model of diversification that predicts that diversification is primarily a function of time and space.

**Results:**

The *Blepharoneura* phylogeny showed more cladogenic divergence associated with geography than with shifts in host-use. Shifts in host-use were associated with ~ 20% of recent splits (< 3 Ma), but > 60% of older splits (> 3 Ma). In the overall tree, gamma statistic and maximum likelihood model fitting showed no evidence of diversification rate changes though there was a weak signature of slowing diversification rate in one of the component clades.

**Conclusions:**

Overall patterns of *Blepharoneura* diversity are inconsistent with a traditional explanation of adaptive radiation involving decreases in diversification rates associated with niche-overlap. Sister lineages usually use the same host-species and host-parts, and multiple non-interbreeding sympatric populations regularly co-occur on the same hosts. We suggest that most lineage origins (phylogenetic splits) occur in allopatry, usually without shifts in host-use, and that subsequent dispersal results in assembly of communities composed of multiple sympatric non-interbreeding populations of flies that share the same hosts.

**Electronic supplementary material:**

The online version of this article (10.1186/s12862-018-1146-9) contains supplementary material, which is available to authorized users.

## Background

How do ecology and geography interact to result in the origin of new animal diversity? From Darwin’s insight that both selection on ecological characters and geographic isolation generate species diversity [1], to Mayr’s [2] insistence that initiation of speciation occurs primarily in the context of geographic isolation, perspectives have shifted as to which factor deserves greater emphasis. Yet, current researchers (e.g. [3, 4]) acknowledge that the roles of ecology and geographic isolation are complementary and obligatorily intertwined. Geography, after all, affects ecology: abiotic factors (soil type, elevation, climate, etc.) vary over geographic space, and distant sites may differ (or not) in their environments. Geographic isolation may therefore result in the accumulation of reproductive isolation due to a combination of factors: stochastic change (e.g.*,* drift), selection in different ecological environments, and sexual selection that can result in divergent patterns of courtship. As individuals from geographically isolated populations move through time and space, secondary contact between previously isolated, divergent populations can allow for ecological interaction between incipient lineages and/or reinforcement of reproductive barriers [5] both of which could result in further evolution of ecologically relevant characters [6].

Exceptions to this synthetic view may often be found among the expansive literature on the evolution of herbivorous insects, a group of animals representing at least a quarter of macroscopic species [7]. For several reasons, research on herbivorous insects has tended to emphasize the importance of ecology in driving speciation. First, herbivory is a key innovation that has consistently resulted in increased diversification across insect taxa [8, 9]. Second, phylogenies of herbivorous insect lineages often show a pattern indicative of adaptive radiation: diversification correlates with entry into successive, often nested adaptive zones defined by plant chemistry and taxonomy [10–12]. Third, studies of herbivorous insects in the incipient stages of speciation suggest a frequent role for ecological differentiation in driving the evolution of reproductive isolation (reviewed in [13–15]). These points have resulted in an emphasis on the importance of ecology, specifically in the form of host shifts, for initiating divergence and driving species diversification [7, 13–15]. Host shifts are thought to promote diversification when courtship and mating are restricted to the host (thus restricting gene flow between host-specific populations) [16] and when host-specific predators select for shifts to “enemy-free space” [17, 18]. Though few would suggest that geographic isolation has played no role in herbivorous insect diversification, its importance in the literature on plant-insect evolution may have been under represented [19, 20].

Our interest in the causes of herbivorous insect speciation stems from our ongoing, intensive study of a tropical herbivore radiation: a diverse group of cryptic species in the genus *Blepharoneura* (Tephritidae). These true fruit flies, found across tropical America, all feed on plants in the Cucurbitaceae (the squash and melon family), and almost all are specialists on specific plant parts (e.g., male versus female flowers, seeds, or stems) of single species of cucurbits. An especially diverse group of *Blepharoneura* specializes on highly sexually dimorphic plants in the subtribe Guraniinae (*Gurania* and its close relative, *Psiguria)*. Previous phylogenetic study [21] revealed ancient colonization of major cucurbit clades (tribes and subtribes), followed by diversification of specialists, often without shifts in host-use.

Local *Blepharoneura* communities reveal a surprising pattern of niche-overlap. At any given site, many reproductively isolated *Blepharoneura* lineages often use the same specific part of the same species of host-plant. For example, at a single site in Ecuador, we reared six species of *Blepharoneura* from *Gurania spinulosa*: three feed on male flowers, two on female flowers, and one feeds on both flower sexes [22]. From a single individual plant (a male *Gurania spinulosa*), we reared four species of *Blepharoneura*, three of which were observed courting (and were captured in *copula*) on the same individual plant. We discovered an even more diverse community along a 1 km transect in Peru: we reared fifteen species of *Blepharoneura* from flowers of just two host plant species: *G. acuminata* and *G. spinulosa* [23], and more recent work suggests this may have been an underestimate of total diversity [24]. Such high levels of niche overlap [22–24] are not predicted by classic models of adaptive radiation [25, 26].

In *Blepharoneura* communities, direct competition for host resources is unlikely: we consistently find that fewer than a third of flowers are occupied by larvae [23]. Furthermore, the current phylogeny shows [21] that many divergence events are not accompanied by host shifts. Those patterns, and the observation that multiple sympatric species occupy the same feeding-niche apparently without competing, led us to suggest that diversification of *Blepharoneura* appeared to be ecologically neutral [21]. Yet, selective pressures associated with host-use are not only associated with competition for food resources. Parasitoids can impose strong selection on patterns of host-use by herbivores [17, 18, 27, 28]: many parasitoids find and attack their hosts only on one species of plant; herbivores on alternate hosts escape death. *Blepharoneura* are attacked by host-specific parasitoid wasps in the opiine subgenus *Bellopius* (Braconidae): each species of *Bellopius* attacks flies in a single host-plant species and sex-flower, and can kill only one species of *Blepharoneur*a [23]. These lethal wasps show greater fidelity to host plant species than even their host flies do, and thus could exert significant selection for shifts to alternate host plants (e.g. [29, 30]).

Lineage-through-time (LTT) plots have been used to identify patterns consistent with adaptive radiation [31–33]. For instance, a classic signature of adaptive radiation is an initial increase in diversification rate, followed by a decrease in diversification rate towards the present (negative gamma values) [25, 26, 32, 34]. This expectation is based on the widely held assumption that speciation and extinction rates are strongly dependent on the number of available, unoccupied niches [26]. Alternatively, ecologically neutral diversification can result in two types of LTT plots. LTT plots with slower initial diversification followed by a rapid accumulation of recent lineages (positive gamma values) are expected when new lineages have a high probability of extinction, as may occur, for example, in lineages that diverge rapidly in response to intense sexual selection (often in allopatry) without ecological divergence [32]. Simulations of metacommunity dynamics [32] suggest that divergence in such lineages can result in multiple cryptic sympatric species having extreme niche overlap (as in *Blepharoneura*). Positive gamma values are also predicted by Hubbell’s neutral theory [35] as a result of the demographic assumption of low initial population size of incipient lineages. LTT plots with a constant diversification rate (gamma ≈ zero) suggest that diversification is a function of time and space, without ecological limits on clade size, and without significant extinction.

To test the hypothesis that ecologically neutral divergence over time and space explain diversification of *Blepharoneura*, we generate a time-calibrated phylogeny that will reveal: 1) patterns in phylogenetic splits associated with changes in host use and/or geographic distribution over time; 2) changes in diversification rate over time. We explore what these patterns can tell us about the processes that have generated and shaped *Blepharoneura* diversity.

## Methods

### Data collection and initial tree inference

**Fig. 1:**
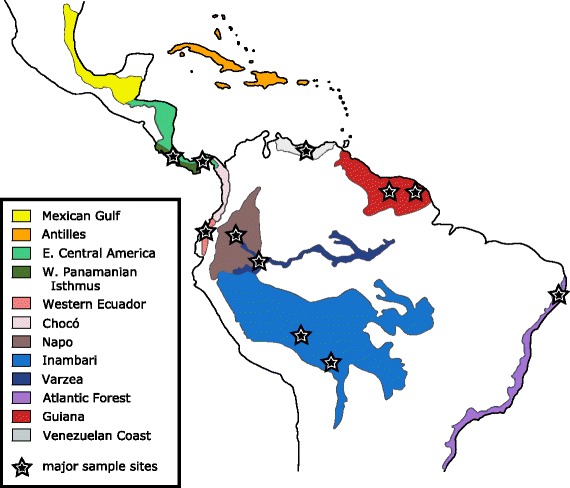
Geographic zones of the Neotropical region as defined in this study, based mostly on Morrone [47], with major sample sites indicated. The “Inambari” zone, as shown, is based on a combination of sources [21, 48]. The two sites in the Western Ecuador and Chocó regions are grouped for our analyses

We used methods described by Condon et al. [21–23] to collect and rear specimens from multiple localities across the Neotropical region (Fig. 1). Our analyses include collections previously reported by Condon et al. [21–23], and specimens from our more recent collections from Costa Rica, Panama, Peru, Bolivia, Brazil, Suriname, and French Guiana (Fig. 1; see also [24]).All specimens were collected, exported, and imported in adherence to national and international regulations, under permits issued by the relevant national agencies (see Additional file 1). Following methods described in Condon et al. [21, 23] we used PCR amplification and sequencing of the mitochondrial cytochrome oxidase 1 (mtCOI) gene (504 bases near the 3′ end) from adult and pupal flies using one of two sets of primers: a set of generalized primers used for adult flies (C1-J-2183/TL2-N-3014; [36], see [21]) or a set of fly-specific primers used for pre- or post-emergence puparia that avoids amplification of fragments from hymenopteran parasitoids (BpupCO1F/BpupCO1R; [23]). As in previous studies, polymerase chain reaction (PCR) amplifications were carried out with a Tetrad 2 thermocycler (Bio-Rad, Hercules, CA, USA) with the following “touchdown” program: initial denaturation for 2 min at 92 °C, 12 touchdown cycles from 58 °C to 46 °C (10 s at 92 °C, 10 s at 58–46 °C, 1.5 min at 72 °C), 27 cycles of 10 s at 92 °C, 10 s at 45 °C, 1.5 min at 72 °C, and a final extension for 7 min at 72 °C. PCR products were cleaned for sequencing using ExoSAP-IT (Affymetrix, Santa Clara, CA, USA) or gel purification using the QIAquick PCR purification kit (Qiagen, Valencia, CA, USA).

The full set of previously published and new mtCOI sequences was collected (3761 total sequences), trimmed and aligned in Geneious v 8.1.8 (Biomatters Ltd.), then imported into R using the APE package [37]. Simple pairwise distances using the number of differences (N) were computed using the dist.dna function in APE, then used to compute a neighbor joining tree in APE.

### Construction & analysis of exemplar data set

Our goal in this study is to understand patterns of *Blepharoneura* diversification across multiple time scales and geographic dimensions. Our previous work [21–23] used 4% mtCOI divergence as a conservative but arbitrary cut-off to define “species.” Analyses of allozymes [38] and microsatellites [24] of sympatric populations show that members of groups (“species”) differing by 4% mtCO1 do not interbreed. Furthermore, microsatellite data [24] clearly reveal non-interbreeding ecologically distinct sympatric populations that differ by less than 4%. Because we are interested in the process of diversification (divergence), which may not always result in speciation (which is difficult to document when divergent populations are allopatric), we chose here to focus on divergent monophyletic “haplotype lineages” differing from one another by at least three mutations (0.5% divergence). This low threshold was chosen to include as much information as possible about the most recent shifts in host use and distribution. Our analyses included an exemplar from each of these lineages. We argue that because many of these haplotype lineages correspond to observed variation in host plant or regional distribution they represent potentially independent evolutionary units.

Nevertheless, to correct for the possibility that some divergent haplotype lineages belong to actually- or potentially-interbreeding populations, analyses were repeated using a truncated phylogeny, derived by removing lineages arising later than 1 million years ago (mega-annum, Ma), then shortening terminal branch lengths by 1 Ma. With few exceptions, analyses of intra-lineage population structure [24] and their strong geographic structure corroborate the assumption that mtCOI haplotype groups at the 1 Ma level of divergence (about 2%) are representative of real, independently evolving biological units. Both ecological and behavioral data also support the assumption that haplotype lineages at the 2% level of divergence reflect evolutionarily relevant units. For example, microsatellite data [24] show that “species 21” reared from different host species represent two distinct, non-interbreeding sympatric populations. Courtship displays differ dramatically between Venezuelan and Ecuadorian (Napo) populations of “species 4” differing by < 2% mtCOI [22, 39].

**Fig. 2:**
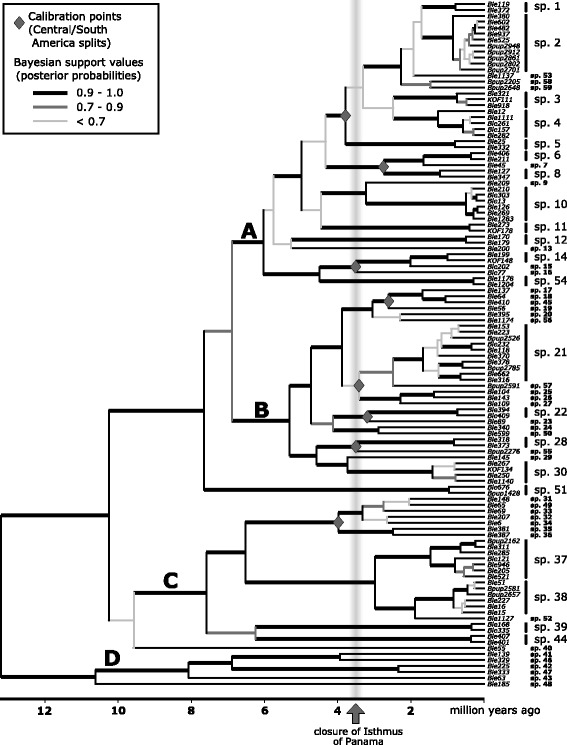
Time calibrated BEAST phylogeny of *Blepharoneura* based on 504 bases of mitochondrial COI from exemplars showing at least 0.5% raw mtCOI divergence, combined with the three gene data set of Condon et al. [21]. The topology shown is a majority rule consensus of post-burnin BEAST trees, with median branch lengths from the BEAST analysis, using a coalescent prior on branch lengths and an unpartitioned dataset. Calibration was imposed as normal priors (mean = 3.5 Ma, sd = 1 Ma) applied to stem groups of eight Central American lineages (indicated by diamonds). Posterior probability branch supports are indicated by branch shade and thickness. “Species” designations at right represent clusters of less than 4% raw mtCOI divergence; spp. 1–49 correspond to those in Condon et al. [21]. Clades are labelled A-D to facilitate discussion in the text

We used the full neighbor joining tree and distance matrix to identify exemplars for a final data set. To do this, we first identified discrete monophyletic lineages on the full tree, circumscribed such that at least three mutations (0.5% divergence) separated exemplars from each lineage. For some clades with complex structure, haplotype networks were constructed using Network v4.6 (Fluxus Engineering) to clarify lineage assignment. In a very limited number of cases the network and neighbor joining structures were such that haplotypes differing by more than three mutations could not be divided into reciprocally monophyletic groups. If this lineage paraphyly involved a single haplotype inferred as ancestral to multiple, divergent haplotypes, and represented by three or fewer specimens, the ancestral haplotype was excluded from further consideration. In two cases where more extensive paraphyly was indicated, descendant lineages plus ancestral haplotypes were combined to form one monophyletic lineage. We then chose exemplars from each of 115 identified haplotype lineages to construct the final data set (Additional file 2: Table S1), including the exemplar sequences of each of 49 “species” defined by > 4% COI divergence and shown in Condon et al. ([21]: Fig. 2; including 6 lineages with only COI data shown in figs. S3-S8 of this reference). The resulting COI alignment was then merged with the multigene alignment of Condon et al. [21], which included COI and the nuclear markers CAD and EF1-α for 43 exemplars. The analysis was rooted with two outgroups from the *Blepharoneura femoralis* group, taken from the Condon et al. [21] data set.

The final merged data set was subjected to Bayesian inference using BEAST v1.8.3 [40]. The BEAST analysis was unpartitioned with a single Markov chain Monte Carlo (MCMC) chain, and used the general time-reversible (GTR) model with four rate categories, an uncorrelated lognormal relaxed clock [41], and a coalescent tree prior. To calibrate the BEAST tree, eight separate Central American clades were identified (two of which included one species from western Ecuador, assumed to be the result of secondary dispersal). A normal constraint with a mean of 3.5 Ma and standard deviation of 1 Ma was imposed on the stem group of each of these eight clades, consistent with the assumption that each represents dispersal subsequent to the complete formation of the Panamanian Isthmus about 3.5 Ma ([42, 43], but see [44]). The analysis was run for ten million generations sampled every 1000 generations, with the first 1 million generations discarded as burnin. Analysis of the run log in Tracer v.1.6 [45] confirmed that stationarity was reached within the burnin period. We computed a majority rule consensus of all sampled post-burnin trees using PAUP* 4.0b10 [46] to represent and summarize results of the BEAST analysis. This consensus tree was then evaluated against the BEAST tree sample for posterior probability branch support and median branch lengths using TreeAnnotator v1.8.4 (part of the BEAST package). After pruning outgroup taxa, the resulting phylogeny and branch lengths were then used in following analyses of character evolution and diversification. To compare inferred node ages and test the effect of these constraints, a separate BEAST analysis was run with the ingroup age only constrained to match the mean inferred from the first analysis (mean = 13.0 Ma, stdev = 0.1 Ma). Three additional BEAST analyses were also run using a Yule prior on branch lengths, a birth-death prior on branch lengths, and with the data set partitioned by gene, in order to test the sensitivity of the analysis to these assumptions.

### Character mapping

In order to investigate temporal patterns of host shift and dispersal and their relationship to cladogenesis, we reconstructed ancestral states for host species, host tissue used, and geographic region. In doing so, we compared multiple methods of character coding and reconstruction to obtain a robust picture of host shifts and dispersal on the *Blepharoneura* history. Host plant information (host species and tissue used) and biogeographic region were noted in an R data frame for each of 3761 specimens identified by mtCOI data, including previously published samples. Geographic regions were designated largely following Morrone [47], with the exception that sites in Bolivia and southern Peru were grouped into a single region (“Inambari”; see e.g. [48]), following Condon et al. [21]. Furthermore, the two sites in Western Ecuador, considered to be in distinct biogeographic regions (Chocó and Western Ecuador), were grouped for our analysis.

Characters were first mapped on the BEAST phylogeny, with outgroups pruned, using the parsimony criterion in Mesquite (v3.03). In the course of our extensive sampling across potential hosts and geographic regions, significant variation was encountered, resulting in some ambiguity in character coding for both host and region, especially when low levels of alternative host use were noted. For example, 90% of specimens belonging to a lineage may be found feeding on a single host species (or a single flower sex), with just a few individuals feeding on alternative hosts. In most of these cases, it is difficult to know if this variation represents real genetic polymorphism, plasticity in host use, or occasional “mistakes” in oviposition. Our fine-scale sampling of exemplars, with inclusion of multiple haplotype lineages for many 4% “species” minimized this problem, as many lineages at this scale were > 95% specific to host and to region. To evaluate the sensitivity of our results to analysis details, we coded within-lineage character variation in host and region in two different ways. This first coding (“polymorphic”) included all states observed for at least 5% of specimens, resulting in a higher proportion of lineages coded as polymorphic. The second coding (“predominant”) included the most frequent state for each lineage, as well as any other states with frequencies within 0.2 of the frequency of the most frequent state. The two coding schemes together represent extremes in the range of possible interpretations of within-lineage variation, and are thus representative of the range of possible evolutionary histories resulting from these interpretations. Host tissue used was coded in an identical fashion, although a greater level of within-lineage variation resulted in more ambiguity in reconstruction for this character.

The second approach we used was stochastic character mapping [49], as implemented in the phytools R package [50]. We input matrices representing the proportion of samples assigned to each lineage that were collected from each region, host and tissue, along with a sample of 250 trees randomly chosen from the BEAST tree sample. These are used to estimate a rate matrix representing the likelihood of evolutionary transitions between each of the possible pairs of character states for each individual tree. A Bayesian MCMC process is then used to sample simulated tip states and character histories that are consistent with the given data. As a stochastic method, this method makes it possible to account for uncertainty in phylogeny reconstruction (by using multiple BEAST trees), within-lineage variation in tip character states (by using raw frequencies of states as a Bayesian prior), and ambiguity in character state reconstruction (by sampling multiple reconstructions).

We used both parsimony reconstruction and stochastic character mapping results to assess the hypothesis that the proportion of lineage splitting events associated with both host shifts and dispersal events have declined over time. To do this, we tallied splits in the region, host, and tissue character maps as occurring before or after a specified breakpoint (3 Ma) and as involving a shift in character state or not. Following Winkler and Mitter [11], a shift was inferred at a given node if nodes or tips representing the two descendant lineages were inferred to have different character states from each other (or non-overlapping sets of states for polymorphic tips). The breakpoint we used (3 Ma) was chosen because it approximates what appears to be an abrupt discontinuity in the proportion of splitting events accompanied by host shifts. Because *Blepharoneura* lineages (and their parasitoids) differ not only in host species used, but also host tissue, a biologically significant shift could involve a change in either of these characters. Accordingly, we counted a shift in either host or tissue as constituting a host shift when tallying results. When parsimony reconstruction of specific ancestral states was ambiguous, we used PAUP* 4.0b10 [46] to assign ancestral states using both accelerated transformation (ACCTRAN) and delayed transformation (DELTRAN; [51]). Results from these two methods were tallied separately to represent the temporal extremes of the possible timing of character transformations. Additionally, we investigated whether patterns varied across the phylogeny by analyzing splits separately for clades A, B and C.

Simmap results were tallied for each of the 2500 simulated reconstructions and summarized using a custom R script. This process was more straightforward, as individual simulations contained no polymorphic or ambiguous character states. As the simmap method is likelihood-based, it accounts for the possibility of multiple, unseen character transitions along branches, and these become almost inevitably inferred in the longest branches. In order to make the simmap results more directly comparable to parsimony mapping, which does not account for branch length, a shift was denoted for a particular splitting event if the two descendent branches exhibited different character states immediately prior to subsequent splitting events, regardless of the number of transitions along internal branches.

Tallies from both the parsimony reconstruction and simmap simulations were summarized graphically, focusing on the difference between the proportions of splits associated with character shifts in older versus younger splits. Tallies were performed both with all splits included, and also with only splits older than 1 Ma included. In order to demonstrate that observed differences do not reflect methodological bias, we conducted a permutation test in R by randomly shuffling the order of rows in a simplified character state matrix with polymorphism eliminated by representing only the most frequent state. We reconstructed parsimony ancestral states using the mpr function of the APE package [37] for the actual character state matrix, then for each of 1000 permuted matrices and compared observed versus permuted differences in split proportions between older and younger splits using an R script.

As an alternative approach to testing for temporal variation in the rate of character change (e.g. host shifts), we used the fitDiscrete function in the GEIGER R package [52] to fit an equal rates likelihood model to the simplified host plant character matrix and phylogeny, then compared the resulting Aikake Information Criterion (AIC) score to those obtained using branch lengths transformed following the early burst model [53] and Pagel’s delta test [54].

### Diversification analyses

We constructed a lineage-through-time plot for the BEAST phylogeny using the ltt.plot function in the APE R package [37], and did the same for the three major clades identified. We then calculated the gamma statistic [31] for the overall BEAST phylogeny and the three clades, using the gammaStat function in APE. Because gamma values are known to be extremely sensitive to lineage sampling [55], especially of near recent lineages [56], we recalculated gamma statistics using the truncated phylogeny. As an alternative test of slowing diversification, we used the fitdAICrc function in the R package laser [57] to fit two constant-rate models (pure birth Yule model and birth-death model) and two density-dependent models representing slowing diversification (DDX: exponential density dependent and DDL: logistic density dependent) to the distribution of branching times [58]. This procedure was also repeated using the truncated phylogeny. For cases where slowing diversification was inferred (negative gamma value or lower AIC scores for density dependent models), the significance of these values and their sensitivity to incomplete taxon sampling was assessed by simulating phylogenies under a Yule (pure birth) model, pruning random tips, then comparing the distribution of the statistic (gamma value or ΔAIC) for simulated trees against values for the actual phylogeny. For each of these simulations, 10,000 trees were simulated using the rphylo function of the APE package [37] with the number of tips set to match the original phylogeny (or truncated phylogeny, or appropriate subclade) after randomly pruning a set percentage of tips (initially 0%; then 10%, 20%, etc.). This percentage was increased until a non-significant result at α = 0.05 was obtained.

## Results

Analysis of available mtCOI sequences identified 115 lineages of *Blepharoneura* differing by at least 3 substitutions (0.5% divergence; Additional file 2: Table S1). At the level of 4% divergence, we found 59 total lineages, which we designated species 1–59, with species 1–49 corresponding to designations in Condon et al. [21]. The BEAST phylogeny (Fig. 2) recovered the four main clades discovered in Condon et al. [21], labelled A-D on Fig. 2.

Comparison of the node age posteriors for Central/South American splits using Tracer [45] in the fully constrained BEAST analysis and the analysis with only the ingroup age constrained showed nearly identical ages (differing by only 0.1 Ma on average) for corresponding nodes, indicating that it is unlikely that one or two erroneous constraints (e.g. dispersal later or earlier than closure of the Panamanian Isthmus) on node ages could have biased posteriors for node ages. Likewise, use of coalescent, Yule, or birth-death priors on branch lengths did not have a noticeable effect on split frequencies or inferred node ages; the same was true of partitioned vs. non-partitioned analyses (Additional file 3: Figure S1).

**Fig. 3:**
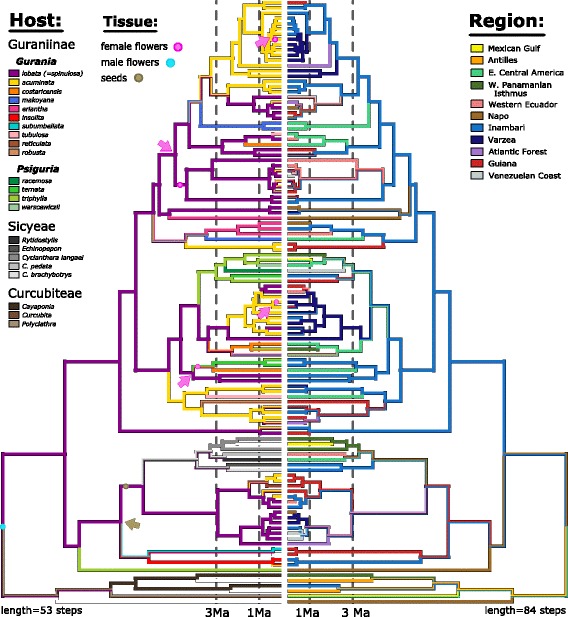
Ancestral character state parsimony reconstruction of host species (left) and geographic region (right) on the BEAST phylogeny. Character states summarize records of all specimens identified by mtCOI sequence, with all states represented by > 5% of total specimens shown on the tips. Inferred ancestral states for host tissue are shown by colored dots on branches, with shifts to feeding on seeds (tan) or specialization on female flowers (pink) shown by colored arrows (several lineages feeding on both flower sexes, with > 5% of specimens on male flowers, are not marked). The dashed lines at 3 Ma divide each tree into older and younger splits, corresponding to the analysis shown in Fig. 4

**Fig. 4:**
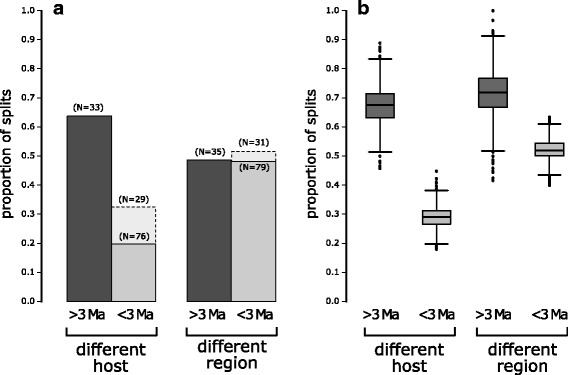
Proportion of lineage splitting events characterized by reconstructed host shifts (i.e. shifts in either host species or host tissue used) and by reconstructed dispersal events within different time periods. Host shift and region are not exclusive: a split may be associated with both host shift and allopatry, one of these, or neither. **a** Proportions based on parsimony reconstruction on the majority rule BEAST tree (Figs. 2 and 3), with states represented by > 5% of total specimens considered. Tips are recorded as different if they do not overlap in character state; DELTRAN optimization was used to resolve reconstruction ambiguity at internal nodes. Dashed lines indicate proportions of splits > 1 Ma (truncated tree). The number of splits in each category is listed above each bar. **b** Boxplots summarizing distribution of proportions found in 2500 stochastic character mappings simulated on 250 randomly chosen untruncated BEAST trees (see Additional file 3: Figure S3 for results based on truncated tree). Observed frequencies of each character state among identified specimens were input as Bayesian priors for each simulation

Visual inspection of results for parsimony reconstruction of ancestral states (Fig. 3) shows that splits associated with distributions in biogeographic regions are more common than splits associated with shifts in host species use, and this is borne out by respective tree lengths (53 steps for host vs. 84 for biogeographic regions, using the polymorphic coding). Using the parsimony reconstructions to tally host shifts versus regional dispersal by split age (Fig. 4a), we found a much lower proportion of recent splits associated with host shifts (20%) than of splits prior to 3 Ma (64%). Proportions of shifts between geographic regions were nearly identical in old and young splits (~ 48%). These trends are not much affected by excluding splits younger than 1 Ma (31% of recent splits associated with host shifts; Fig. 4a; Additional file 2: Table S2). Proportions of splits associated with host or geographic shifts varied slightly under different coding schemes and reconstructions (Additional file 2: Table S2), but all show a similar difference between old versus young splits for host shifts, and also a similar lack of differentiation for geography. This general pattern held true for each clade individually, though proportions differed somewhat (Additional file 2: Table S2). Likewise, stochastic character mapping (Fig. 4b) showed proportions similar to parsimony for host, but in contrast inferred a slightly higher proportion of geographic shifts for older splits compared to younger ones. Results of the permutation analysis (Additional file 3: Figure S2) confirmed that the observed difference in proportion of old versus young splits accompanied by host shifts falls well outside the simulated distribution (*p* = 0). In contrast to these observed differences in proportion of splits with host shifts, model fitting in GEIGER found that early burst [53] and Pagel’s delta [54] models did not show a better fit to observed host use character states than a simple, constant rate model (ΔAIC = + 1.69 for early burst model, + 1.66 for delta model).

**Fig. 5:**
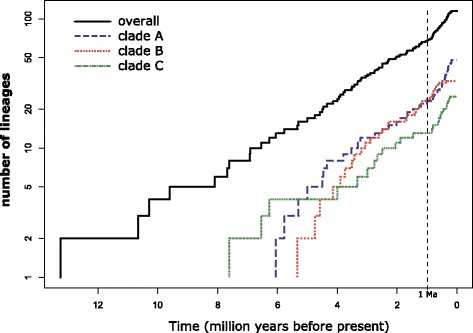
Lineage-through-time plots for the overall phylogeny (black, above), and three component clades. Clade A includes sp. 1–16, clade B includes sp. 17–30, and clade C includes sp. 31–39, with some newly designated lineages included in each of these. The dashed line at 1 Ma indicates point of truncation (for truncated trees). Clade B shows a significant slowdown in diversification rate (γ = 2.09, *p* = 0.037), as measured by the γ statistic of Pybus and Harvey [31]

The LTT plot for the overall phylogeny (Fig. 5) shows a relatively steady rate of diversification, and this is corroborated by a non-significant positive gamma value (γ = 1.45; values over 1.96 are significant at the level of alpha = 0.05 for a two-tailed test; see [31]). Patterns revealed by individual clades differ from the overall pattern (Fig. 5). Clades A and C show positive but non-significant gamma values (γ =1.21 for A; γ =1.18 for C), but Clade B shows an overall decrease in diversification rate over time, as shown by a significantly negative gamma (γ = − 2.09). Simulations show that the negative gamma of Clade B remains significant even if 20% of taxa are assumed to be missing from the phylogeny; however, significant differences among the individuals clades disappear in the truncated phylogeny. In the truncated phylogeny, which only includes lineages that split ≥ 1 Ma, we find no significant variation from γ = 0, indicating a relatively constant rate of diversification (Table 1).

**Table 1:** Gamma statistics and associated *p* values (two-tailed) for *Blepharoneura* and various subclades

Clade	Lineages	γ	*p*	Max % missing (2-tailed)
All clades	all	1.45	0.147	ns
Clade A	all	1.21	0.225	ns
Clade B	all	−2.09	0.037	20%
Clade C	all	1.18	0.237	ns
All clades	> 1 Ma	−0.348	0.36	ns
Clade A	> 1 Ma	−1.25	0.11	ns
Clade B	> 1 Ma	−0.88	0.19	ns
Clade C	> 1 Ma	−0.45	0.33	ns

Top four rows, “all” lineages = lineages differing by ≥ 3 observed mutations in mtCOI. Bottom four rows: results of analyses of tree truncated at 1 Ma. A significantly negative gamma indicates slowing of diversification near the present, and is most strongly affected by the most recent splits. *P* values shown assume complete taxon representation, calculated directly from the gamma statistics as shown by Pybus and Harvey [31]. The maximum percent missing column shows the highest percentage of missing taxa (in 10% increments) for simulations in which adjusted critical values for **γ** were higher than the **γ** value shown. ns indicates that the gamma value was not significant compared to the simulated null distribution even assuming complete taxon sampling

Fitting maximum likelihood models of diversification in LASER gave results similar to gamma statistics (Table 2). In analysis of the full tree (115 terminals differing by > 0.5% mtCOI), Aikake Information Criteria values were lowest (i.e., showed best fit) for constant-rate models. When the analysis included all lineages (Clades A-D), a birth-death model showed the best fit; when clades were analyzed separately, a pure birth model was the best fit for clades A and C (Table 2). Clade B showed a significantly better fit to a density dependent model (DDL), indicating a possible slowdown in diversification. Simulations, however, showed that the fit to DDL loses significance if greater than 10% of lineages are assumed to be missing (Table 2). When using the phylogeny truncated at 1 Ma, simulations showed no significant difference from constant rate models (Yule, pure birth model) in one-tailed tests of the DDL/DDX models.

**Table 2:** Aikake Information Criterion (AIC) values from fitting models of diversification rate using the fitdAICrc function of LASER [57, 58]

Clade	Lineages	Best fitting model	Max % missing (1-tailed)
All clades	all	birth-death	n/a
Clade A	all	pure birth	n/a
Clade B	all	DDL	10%
Clade C	all	pure birth	n/a
All clades	> 1 Ma	pure birth	n/a
Clade A	> 1 Ma	DDX	ns
Clade B	> 1 Ma	DDX	ns
Clade C	> 1 Ma	pure birth	n/a

Top four rows, “all” lineages = lineages differing by ≥ 3 observed mutations in mtCOI. Bottom four rows: results of analyses of tree truncated at 1 Ma. Constant rate models with (birth-death) and without (pure birth) extinction were compared to logarithmic (DDL) and exponential (DDX) density dependent models in which diversification rate declines as a function of the number of lineages. The “best fitting model” column identifies models with the lowest AIC value. The ΔAIC column indicates the difference in AIC values between the best fitting density dependent model and the best fitting constant rate model, such that positive values indicate a better fit to density dependent models. The maximum percent missing column shows the highest percentage of missing taxa (in 10% increments) for simulations in which critical values for ΔAIC values were lower than the observed ΔAIC value shown. NS indicates that the ΔAIC value was not significant even assuming complete taxon sampling. N/A indicates “not applicable” because the test was a one-tailed test

## Discussion

Models of adaptive radiation suggest that the rate of diversification, as well as rates of host-shifts, will increase as lineages invade new ecological niches, and will slow down as niches are filled [1, 25, 26]. Much work on herbivorous insects suggests that shifts to new host-plant niches drive diversification [7–15]. Our results, however, show that the proportion of splits associated with host-shifts is lower among recent lineages of *Blepharoneura* (Fig. 4), many of which occupy the same niches, but diversification rates do not slow down (Fig. 5; Tables 1 and 2). Instead, as lineages continue to diversify, patterns of host-use are generally conserved, and recent diversification (0-3mya) is primarily associated with geographic isolation (Fig. 4).

We have evidence (here and elsewhere [24]) that initial genetic divergence in *Blepharoneura* often reflects geographic dispersal and isolation. Ancestral state reconstruction of geographic region on the phylogeny (Fig. 3) shows that about half of all lineage-splitting events, both recent and ancient, involve dispersal to different bioregions. Microsatellite variation in widespread species also indicates a prominent role for geographic isolation [24]. For example, analyses of microsatellites indicate that many lineages show strong signatures of isolation across the Amazon Basin, such that populations in Venezuela, Suriname, and French Guiana are genetically distinct from populations in Bolivia and Peru. Geographic isolation at finer scales is also likely. For example, some lineages show microsatellite variation among sites separated by less than 100 km [24]. Most sympatric genetically distinct populations that use different hosts are not one another’s closest relatives; instead, sister lineages are found in other geographic regions, indicating that divergence initiates in allopatry and host shifts occur later. Dispersals across the Panamanian Isthmus represent a special case: because there is little species-level overlap in the cucurbit assemblages in Central vs. South America, populations dispersing to Central America necessarily underwent simultaneous host shifts to plants available in the new area.

Microsatellite data [24] also suggest that splits associated with host plant shifts in allopatry may increase the likelihood of lineage persistence upon secondary contact. This is suggested by the observation that no two lineages below the 4% mtCOI sequence divergence level with distinct microsatellite allele frequencies were detected coexisting on the same host species and tissue at a single site; mtCOI lineages that coexist on single hosts are not sister taxa. We suggest that most lineages diverge in allopatry, and that upon secondary contact, divergent lineages on identical hosts experience one of three outcomes:

First, genetically distinct lineages using the same hosts may merge because isolating barriers may be insufficiently strong to maintain reproductive isolation. Second, reinforcement – selection against hybridization between divergent, reproductively isolated lineages – may favor host shifts upon secondary contact if a host shift reduces encounters between flies of different lineages. Third, upon secondary contact, lineages may become extinct. Extinction could simply be due to stochastic factors - especially if new lineages have small population sizes - or extinction could occur if individuals from previously allopatric populations are more susceptible to parasitoids in a newly invaded community. Parasitoid mediated extinction may be less likely following a host shift, because *Bellopius* parasitoids show high specificity to plant host [23].

The strong signal of allopatry in our data has direct implications for sampling in this and similar studies. We found that much of the genetic variation in *Blepharoneura*, as in many organisms, corresponds to genetically divergent geographic isolates whose future fate is unknown. Although these could be considered intermediate stages in the protracted process of speciation, many of them will presumably go extinct or merge upon contact with other populations. It is thus premature to classify most of these geographic isolates as biological species [59], yet they represent relevant units in the ongoing diversification of *Blepharoneura*. For this reason we deliberately chose a low threshold of divergence (0.5% mtCOI divergence) for identifying samples to include in our analyses. We then repeated analyses with a phylogeny truncated at 1 Ma to test the sensitivity of our results to thresholds for sampling recent lineages. Our inference of constant diversification rate did not change between these sets of analyses, showing that inclusion of lineages at a low divergence threshold did not bias our results. In contrast, thanks to comments from reviewers, we discovered that the use of typical cutoff values (~ 2%; [60]) for lineage delimitation results in a specious inference of slowing diversification, for both the gamma statistic and maximum likelihood model comparisons. The same may be true when considering only taxonomically described morphospecies without including genetically divergent, cryptic species.

Our results show a strong decrease in the proportion of divergence events associated with host-shifts in the near-present (< 3 Ma; Fig. 4), using both the full and truncated phylogeny. A similar result was noted by Nyman et al. [20], who found that about 50% of phylogenetic splits in their sample of nematine sawflies were associated with differences in host taxon, feeding mode, or tissue used. By applying a logistic regression model to the host shift data, Nyman et al. [20] showed that the most recent splits had a much lower probability (about 20%) of exhibiting niche differences. Although early bursts of trait evolution are consistent with theories of adaptive radiation, such signatures are rare in actual phylogenies [53] and should be accompanied by slowing diversification, which we did not observe. We suggest instead that ecologically mediated persistence of lineages (e.g., via host shifts to enemy-free space) could explain why we see a greater proportion of splits associated with host shifts among deep splits than among recent splits (Fig. 4). If extinction is less likely after shifts to novel hosts, and most splits do not involve host-shifts, then the excess of lineage splitting events without host shifts in the very recent may largely represent transient lineages that are fated for extinction.

The two major clades of *Blepharoneura* (A and B) differ somewhat in their observed rates of host-shift. Parasitoids of *Blepharoneura*, which are even more highly specific to the flies’ host-plants than the flies are, and flies’ defenses against those parasitoids [23] may help explain this pattern. We advance the preliminary hypothesis that clade-specific differences in flies’ abilities to defend themselves against parasitoids would affect the strength of selection for host-shifts to enemy-free space [17, 18]: selection for host-shifts would be stronger in flies with weak defenses (Clade B). Clade A, which shows a lower frequency of host shifts, includes several lineages (e.g., sp. 1,2,3) with extremely low rates of parasitism, while Clade B, which shows a higher frequency of host shifts (Fig. 3), includes several lineages (e.g., sp. 21, 28) with exceptionally high rates of parasitism [23], which would select for host-shifts. Failure to shift hosts might result in local extinctions, which might explain why Clade B is the only clade that shows some evidence of a slow-down in recent diversification (Fig. 5, Tables 1 and 2). Investigation of this question will require integration of phylogenetic data with data on the functional genomics of *Blepharoneura* and their parasitoids, as well as experimental evidence, but would greatly expand our understanding of how biodiversity arises in complex, multitrophic tropical communities.

Promising future research directions include the use of population genomic data to obtain a more detailed picture of the context and course of speciation, documenting the evolution of courtship behavior and its effect on current patterns of reproductive isolation, and determining the role of parasitoids in mediating host shifts and coexistence or extinction of *Blepharoneura* lineages. If patterns of diversification of *Blepharoneura* are typical for other groups of tropical insects, pure birth models of diversification, without ecological limitation, may largely explain the extraordinary diversity of insects in the tropics.

## Conclusion

A common view of adaptive radiation suggests that species diversity is bounded by an ecologically determined upper limit of species (evolutionary “carrying capacity”; [26, 61]) that causes speciation to slow as available niches are filled. Patterns of diversification in *Blepharoneura* flies, however, are not consistent with ecologically limited models of adaptive radiation. Instead, we find support for constant-rate models of diversification, suggesting that *Blepharoneura* diversity may be “dynamic and unbounded” [62]. Our study further shows that patterns of host-use are highly conserved, and that most lineages diverge in allopatry, without host-shifts. Time and space may largely explain the diversity of this Neotropical genus, and may explain the high levels of insect diversity in the tropics.

## Additional files

Additional file 1: Spreadsheet with list of permits obtained for research, collection, export and import of specimens used in this study, and respective granting agencies. (XLSX 16 kb)

Additional file 2: **Table S1.** *Blepharoneura* lineages identified in the analysis, along with Genbank accession numbers and summaries of host use and distribution based on available mtCOI barcodes. **Table S2.** Proportions of lineage splitting events associated with host shifts (either host species or host tissue) and geographic region by split age. (DOCX 33 kb)

Additional file 3: **Figure S1.** Comparison of inferred node ages for BEAST analyses incorporating different prior settings and assumptions. **Figure S2.** Results of the permutation test showing significance of the difference in the proportion of old versus young splits that are associated with inferred shifts in host or geographic region. **Figure S3.** Summary of stochastic character mapping results for truncated tree. (PDF 205 kb)

## Abbreviations

ACCTRAN: accelerated transformation
AIC: Aikake information criterion
DDL: density dependent-logistic
DDX: density dependent-exponential
DELTRAN: delayed transformation
GTR: general time-reversible
LTT: Lineage through time
Ma: mega-annum (million years before present)
MCMC: Markov Chain Monte Carlo
mtCOI: mitochondrial cytochrome oxidase 1
PCR: polymerase chain reaction
