# Supplementary material for: Anatomy of a Neotropical insect radiation
Source: BMC Evol Biol. 2018 Mar 14;18:30. doi: 10.1186/s12862-018-1146-9 (PMC5853117; doi:10.1186/s12862-018-1146-9)
Supplement: Supplementary file 2 — Table S1. Blepharoneura lineages identified in the analysis, along with Genbank accession numbers and summaries of host use and distribution based on available mtCOI barcodes. Table S2. Proportions of lineage splitting events associated with host shifts (either host species or host tissue) and geographic region by split age. (DOCX 33 kb) [file 12862_2018_1146_MOESM2_ESM.docx]

**Table S1.** *Blepharoneura* lineages identified in the analysis, along with Genbank accession numbers and summaries of host use and distribution based on available mtCOI barcodes.

| **lineage** | **exemplar** | **n** | **Genbank accession #** | **hosts** | **tissue** | **distribution** |
| --- | --- | --- | --- | --- | --- | --- |
| **INGROUP** |  |  |  |  |  |  |
| sp. 1a | Ble119 | 117 | EU601913 | GA(114), GS(3) | m(104), f(13) | GUI |
| sp. 1b | Ble372 | 54 | EU601900 | GA | m(30), f(24) | INA |
| sp. 2a | Ble380 | 25 | EU601889 | GA | m(16), f(9) | INA |
| sp. 2b | Ble602 | 36 | KF473707 | GA(35), GS(1) | m(34), f(2) | INA |
| sp. 2c | Ble482 | 14 | KF473722 | GA | m(13), f(1) | INA |
| sp. 2d | Ble937 | 2 | KF473831 | GA | m | INA |
| sp. 2e | Ble525 | 43 | KF474698 | GA | m | INA |
| sp. 2f | Bpup2948 | 1 | KY312817 | GA | f | VAR |
| sp. 2g | Bpup2912 | 1 | KY312818 | GA | m | VAR |
| sp. 2h | Bpup2861 | 94 | KY312821 | GA(91), GS(2), GE(1) | m(68), f(26) | VAR |
| sp. 2i | Bpup2802 | 2 | KY312819 | GA | m | VAR |
| sp. 2j | Bpup2701 | 3 | KY312820 | GA | m | VAR |
| sp. 3a | Ble321 | 218 | EU601792 | GA(217), GS(1) | m(216), f(2) | INA |
| sp. 3b | KOF111 | 4 | KY312822 | GA | m | INA |
| sp. 3c | Ble918 | 4 | KF474278 | GA | m | INA |
| sp. 4a | Ble12 | 2 | EU601952 | GS | m | GUI(1), VEN(1) |
| sp. 4b | Ble1111 | 72 | KY312823 | GS(66), GA(6) | m | ATF |
| sp. 4c | Ble261 | 4 | EF531814 | GS | m | NAP |
| sp. 4d | Ble157 | 223 | EU602067 | GS(219), GA(4) | m(210), f(13) | GUI |
| sp. 4e | Ble282 | 229 | EU602081 | GS(223), GA(2) | m(220), f(5) | INA(180), NAP(37), VAR(10), GUI(2) |
| sp. 5a | Ble25 | 9 | EU601861 | GM | m | ECA |
| sp. 5b | Ble332 | 17 | EU601851 | GM | f(14), m(1) | ECA |
| sp. 6a | Ble406 | 10 | EU602079 | GT | m(10) | ECA |
| sp. 6b | Ble211 | 7 | EU602011 | GS | m(6), f(1) | WEC |
| **lineage** | **exemplar** | **n** | **Genbank accession #** | **hosts** | **tissue** | **distribution** |
| sp. 7 | Ble45 | 25 | EU602111 | GC | m(22), f(2) | ECA |
| sp. 8a | Ble127 | 108 | EU601945 | GS(103), GA(2) | m(98), f(7) | GUI |
| sp. 8b | Ble347 | 158 | EU601833 | GS(149), GE(2), GA(1) | m(148), f(4) | INA(120), NAP(29), VAR(9) |
| sp. 9 | Ble209 | 6 | EU601992 | GS | m(3), f(3) | WEC |
| sp. 10a | Ble210 | 2 | EU602006 | GS | f | WEC |
| sp. 10b | Ble303 | 29 | EU601787 | GS(27), GA(2) | f(28), m(1) | INA(18), GUI(10), VAR(1) |
| sp. 10c | Ble13 | 2 | EU601919 | GS | f | VEN |
| sp. 10d | Ble126 | 93 | EU601946 | GS(73), Grob(13), GA(7) | f(90), m(3) | GUI(78), INA(8), VAR(7) |
| sp. 10e | Ble269 | 123 | EF531811 | GS(95), GA(26), Grob(2) | f(119), m(4) | GUI(40), INA(33), VAR(27), NAP(23) |
| sp. 10f | Ble1283 | 3 | KY312824 | GS(1), Grob(1) | m(1), f(1) | GUI |
| sp. 11a | Ble273 | 99 | EF531810 | GS | f(98), m(1) | GUI(55), INA(24), NAP(14), VAR(6) |
| sp. 11b | KOF178 | 1 | KY312825 | GS | f | INA |
| sp. 12a | Ble170 | 6 | EF531757 | GS | m | NAP |
| sp. 12b | Ble179 | 45 | EF531823 | GS | m | INA(40), VAR(3), NAP(2) |
| sp. 13 | Ble200 | 6 | EU602001 | GE | m(4), f(2) | NAP |
| sp. 14a | Ble199 | 2 | EU601997 | GE | m | NAP |
| sp. 14b | KOF148 | 1 | KY312826 | GE | m | INA |
| sp. 15 | Ble202 | 2 | EU601998 | GE | m | WEC |
| sp. 16 | Ble77 | 1 | EU602096 | GM | m | ECA |
| sp. 17 | Ble137 | 1 | EU601949 | Ptri | m | WPI |
| sp. 18 | Ble64 | 5 | EU601882 | Ptri | m | ECA(3), MEG(2) |
| sp. 19 | Ble56 | 1 | EU601871 | Prac | m | VEN |
| sp. 20 | Ble395 | 2 | EU601907 | Pter | m | INA |
| sp. 21a | Ble153 | 37 | EU602087 | GS(1), GR(1) | m(35), f(2) | GUI(36), VEN(1) |
| sp. 21b | Ble223 | 2 | EU601985 | GS | m | WEC |
| sp. 21c | Bpup2526 | 24 | KY312827 | GA | m | VAR |
| sp. 21d | Ble232 | 60 | EU601767 | GA | f(59), m(1) | VAR(58), INA(2) |
| sp. 21e | Ble118 | 68 | EU602097 | GA(66), GS(2) | m(43), f(25) | GUI(58), VAR(6), INA(4) |
| **lineage** | **exemplar** | **n** | **Genbank accession #** | **hosts** | **tissue** | **distribution** |
| sp. 21f | Ble370 | 38 | EU601899 | GA(37), GS(1) | m | VAR(28), INA(10) |
| sp. 21g | Ble378 | 1 | EU601894 | GA | m | INA |
| sp. 21h | Bpup2785 | 1 | KY312828 | GA | m | VAR |
| sp. 21i | Ble662 | 65 | KF473831 | GS | m | INA(59), VAR(6) |
| sp. 21j | Ble316 | 36 | EU602016 | GA | f(24), m(12) | VAR(25), INA(11) |
| sp. 22a | Ble394 | 2 | EU601909 | Pter(1), Ptri(1) | m(1), f(1) | INA |
| sp. 22b | Ble409 | 1 | EU601798 | Pter(1) | f(1) | INA |
| sp. 23 | Ble89 | 2 | EU602068 | GC | f | ECA |
| sp. 24 | Ble340 | 4 | EU601820 | GS | m | INA |
| sp. 25 | Ble104 | 28 | EU602101 | GC | m(26), f(2) | ECA |
| sp. 26 | Ble143 | 3 | EU601939 | GC(2), Ptri(1) | m | WPI |
| sp. 27 | Ble109 | 68 | EU602107 | GC(27), GT(25), GM(8), GS(7), Gped(1) | f(41), m(26) | ECA(59), WEC(2) |
| sp. 28a | Ble318 | 125 | EU602021 | GA(123), GS(1) | m | INA(101), VAR(23), GUI(1) |
| sp. 28b | Ble373 | 43 | EU601902 | GA | m | INA |
| sp. 29 | Ble145 | 46 | EU601957 | Grob(35), GR(10), GA(1) | m(44), f(2) | GUI |
| sp. 30a | Ble267 | 583 | EF531800 | GS(483), GA(94), GI(1), GR(1), Gsub(1) | m(347), f(234) | GUI(331), INA(165), VAR(59), NAP(28) |
| sp. 30b | KOF134 | 3 | KX902902 | GA | f(2), m(1) | INA |
| sp. 30c | Ble250 | 8 | EU602099 | GA | m | GUI |
| sp. 30d | Ble1140 | 14 | KY312829 | GA(11), GS(3) | m | ATF |
| sp. 31 | Ble148 | 2 | EU601968 | Cycl | s | WPI |
| sp. 32 | Ble207 | 1 | EU602119 | Cycp | s | WEC |
| sp. 33 | Ble69 | 3 | EU602022 | Cycl | s | WPI |
| sp. 34 | Ble6 | 2 | EU602017 | Ryt | s | ECA |
| sp. 35 | Ble381 | 11 | EU601892 | Erac | s | INA |
| sp. 36 | Ble387 | 129 | EU601826 | Cycb | s | INA |
| sp. 37a | Bpup2162 | 5 | KY312830 | GA | s | GUI |
| **lineage** | **exemplar** | **n** | **Genbank accession #** | **hosts** | **tissue** | **distribution** |
| sp. 37b | Ble311 | 4 | EU601788 | GA | s | GUI |
| sp. 37c | Ble285 | 4 | EU602075 | GR | s | GUI |
| sp. 37d | Ble121 | 5 | EU601955 | GS(4), GA(1) | s | GUI |
| sp. 37e | Ble946 | 2 | KF473870 | GA | s | INA |
| sp. 37f | Ble205 | 4 | EU601992 | GS | s | WEC |
| sp. 37g | Ble521 | 3 | KF474336 | GS | s | INA |
| sp. 38a | Ble51 | 7 | EF531753 | GS | s | NAP |
| sp. 38b | Bpup2581 | 3 | KY312832 | GS | s | VAR |
| sp. 38c | Bpup2657 | 2 | KY312831 | GS | s | VAR |
| sp. 38d | Ble227 | 3 | EU601976 | GS | s | INA |
| sp. 38e | Ble16 | 1 | EU601960 | GS | s | VEN |
| sp. 38f | Ble15 | 1 | EU601958 | GS | s | VEN |
| sp. 39a | Ble168 | 25 | EU601926 | Gsub(24), GA(1) | m(21), f(2), s(2) | GUI |
| sp. 39b | Ble335 | 1 | EU601822 | ? | ? | GUI |
| sp. 40 | Ble55 | 1 | EU602109 | Ptri | m | NAP |
| sp. 41 | Ble139 | 5 | EU601941 | Cay | m | WPI |
| sp. 42 | Ble225 | 3 | EU601966 | Cay | m | INA |
| sp. 43 | Ble63 | 5 | EU601866 | Cuc(4), Pol(1) | m | ECA(3), MEG(2) |
| sp. 44a | Ble407 | 1 | EU601766 | GI | m | INA |
| sp. 44b | Ble401 | 5 | EU602103 | GI(3), GA(2) | m | INA |
| sp. 45 | Ble410 | 1 | EU601765 | Pwar | m | ECA |
| sp. 46 | Ble329 | 2 | EU601865 | ? | ? | ANT |
| sp. 47 | Ble333 | 1 | EU601855 | ? | ? | ANT |
| sp. 48 | Ble185 | 1 | EF531752 | ? | ? | NAP |
| sp. 49 | Ble65 | 1 | EU602117 | ? | ? | MEG |
| sp. 50 | Ble599 | 171 | KF473984 | GS(162), GA(6), GI(3) | m(168), f(3) | INA |
| sp. 51a | Ble676 | 3 | KF474530 | GS(2), GA(1) | m | INA |
| sp. 51b | Bpup1428 | 10 | KY312833 | GS | m(7), f(3) | GUI |
| sp. 52 | Ble1127 | 52 | KY312834 | GS | s | ATF |
| sp. 53 | Ble1137 | 5 | KY312835 | GA | m | ATF |
| sp. 54a | Ble1178 | 3 | KY312836 | GA | m | GUI |
| sp. 54b | Ble1204 | 19 | KY312837 | GA | m(18), f(1) | GUI |
| sp. 55 | Bpup2276 | 3 | KY312838 | GT | m | ECA |
| sp. 56 | Ble1174 | 10 | KY312839 | Ptri | m | GUI |
| sp. 57 | Bpup2591 | 3 | KY312840 | GS | m | VAR |
| sp. 58 | Bpup2205 | 34 | KY312841 | GA(33), GS(1) | m(31), f(3) | VAR |
| sp. 59 | Bpup2648 | 1 | KY312842 | GA | m | VAR |
| **OUTGROUP** |  |  |  |  |  |  |
| *B. furcifer* | Ble416 | 1 | EU602120 | ? | ? |  |
| B. sp. OUT2 | Ble141 | 2 | EU602059 | Sech | stem | WPI |
| sp. 60 | Bpup2089 | 2 | KY312843 | GA | s | ATF |
| sp. 61 | Bpup2097 | 6 | KY312844 | GA | s | ATF |
| sp. 62 | Bpup2576 | 1 | KY312845 | Grhi | m | VAR |

Species numbers correspond to heuristic species designations in Condon et. al [1], based on 4% mtCOI divergence. Exemplars are specimens used in phylogenetic analysis (denoted by extraction codes); the numbers of total specimen barcodes (n) used to infer host and geographic range are listed. If flies were reared from multiple hosts or tissues, or from multiple regions, numbers of specimens from each are noted in parentheses. A small number of specimens were caught as adults and not reared; these are included in total specimen numbers, but not listed in host totals.

**Abbreviations: (host)** GA, *Gurania acuminata*; GC, *G. costaricensis*; GE, *G. eriantha*; GI, *G. insolita*; GM, *G. makoyana*; Gped, *G. pedata*; GR, *G. reticulata*; Grhi, *G. rhizantha*; Grob*, G. robusta*; GS, *G. spinulosa*; Gsub, *G. subumbellata*; GT, *G. tubulosa*; Prac, *Psiguria racemosa*; Pter, *P. ternata*; Ptri, *P. triphylla*; Pwar, P*. warscewiczii*; Cycl, *Cyclanthera langaei*; Cycp, *C. pedata*; Cycb, *C. brachybotrys*; Erac, *Echinopepon racemosus*; Ryt, *Rytidostylis gracilis*; Sech, *Sechium pittieri*; Cay, *Cayaponia spp.*; Cuc, *Cucurbita pepo*; Pol, *Polyclathra cucumerina*; **(region)** MEG, Mexican Gulf; ANT, Antilles; ECA, Eastern Central America; WPI, Western Panamanian Isthmus; WEC, Western Ecuador (including Chocó); NAP, Napo; INA, Inambari; VAR, Varzea; ATF, Atlantic Forest; GUI, Guiana; VEN, Venezuelan Coast; **(tissue)** m, male flowers; f, female flowers; s, seeds.

**Reference:** 1. Condon MA, Scheffer S J, Lewis ML, Swensen SM. Hidden neotropical diversity: greater than the sum of its parts. Science. 2008; 320:928–931.

**Table S2.** Proportions of lineage splitting events associated with host shifts (either host species or host tissue) and geographic region by split age, calculated using different character coding and optimization schemes.

| coding | optimization | lineages | host, >3 Ma | host, <3 Ma | geo, >3 Ma | geo, <3 Ma |
| --- | --- | --- | --- | --- | --- | --- |
| poly | DEL | all | 0.64 | 0.20 | 0.49 | 0.48 |
| poly | ACC | all | 0.64 | 0.21 | 0.60 | 0.48 |
| pred | DEL | all | 0.70 | 0.25 | 0.51 | 0.54 |
| pred | ACC | all | 0.67 | 0.25 | 0.57 | 0.53 |
| poly | DEL | > 1 Ma | 0.64 | 0.31 | 0.49 | 0.52 |
| poly | DEL | clade A | 0.64 | 0.11 | 0.36 | 0.50 |
| poly | DEL | clade B | 0.60 | 0.27 | 0.50 | 0.36 |
| poly | DEL | clade C | 1.0 | 0.27 | 0.60 | 0.53 |

Abbreviations: poly=polymorphic coding (including all states found in >5% of samples); pred=predominant coding (including only the most frequent state, plus those with frequencies within 0.2 of the most frequent state); ACC=accelerated transformation parsimony optimization; DEL=delayed transformation parsimony optimization.
