# Supplementary material for: Anatomy of a Neotropical insect radiation
Source: BMC Evol Biol. 2018 Mar 14;18:30. doi: 10.1186/s12862-018-1146-9 (PMC5853117; doi:10.1186/s12862-018-1146-9)
Supplement: Supplementary file 3 — Figure S1. Comparison of inferred node ages for BEAST analyses incorporating different prior settings and assumptions. Figure S2. Results of the permutation test showing significance of the difference in the proportion of old versus young splits that are associated with inferred shifts in host or geographic region. Figure S3. Summary of stochastic character mapping results for truncated tree. (PDF 205 kb) [file 12862_2018_1146_MOESM3_ESM.pdf]

median node ages

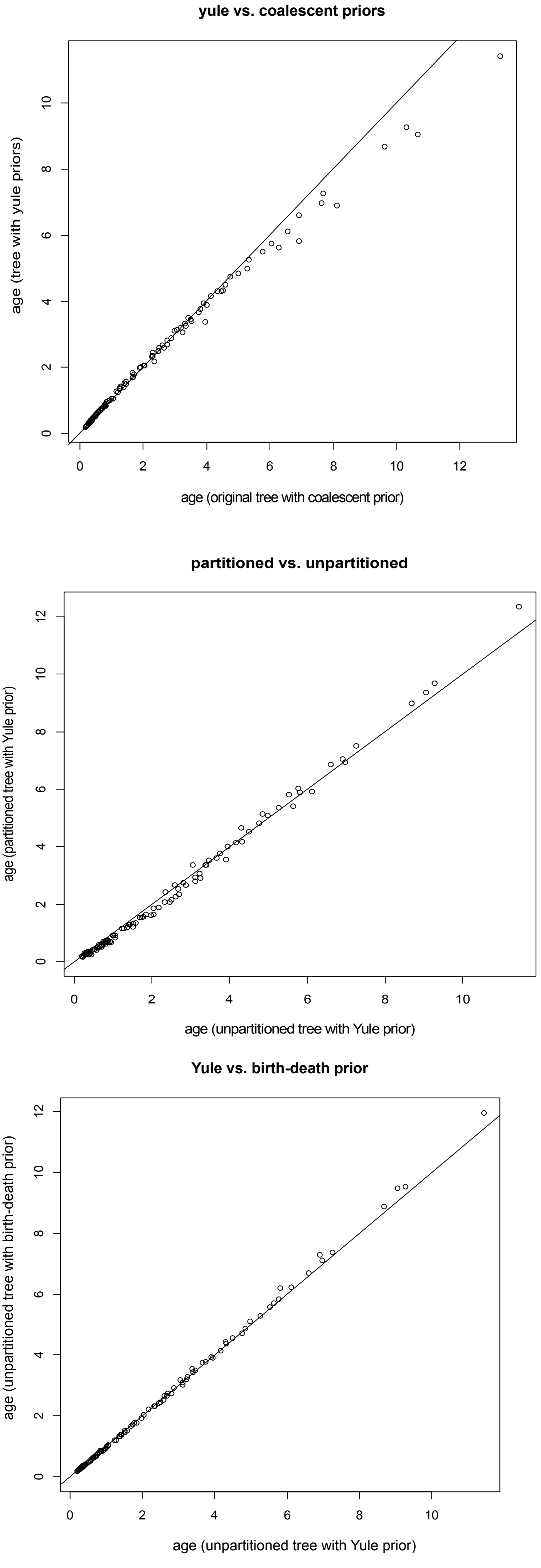

Figure S1. Comparison of median node ages between four BEAST runs with different settings (coalescent vs. Yule or birth-death priors on branch lengths and unpartitioned vs. partitioned by gene), showing minimal differences between runs. The partitioned analysis was run for 100 million generations (necessary for adequate effective sample size for some parameters), while others were run for 10 million generations.

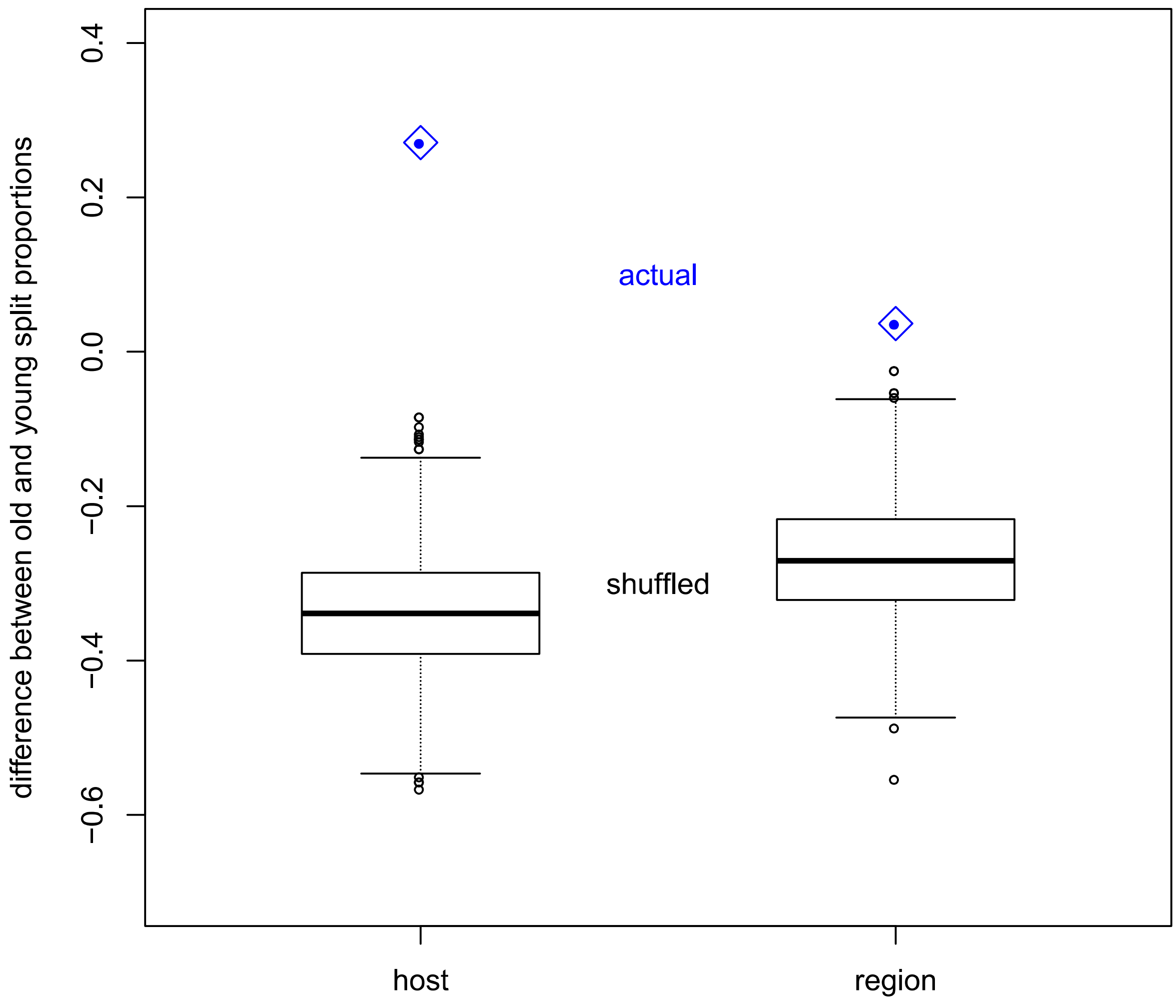

Figure S2. Results of the permutation test showing the difference in the proportion of old (> 3 Ma) versus young (< 3 Ma) splits that are associated with inferred shifts in host or geographic region based on parsimony mapping of character states on the full haplotype lineage phylogeny (actual proportions are denoted by blue diamonds) versus the distribution of the difference based on 1,000 permuted data sets in which character states were randomly shuffled.

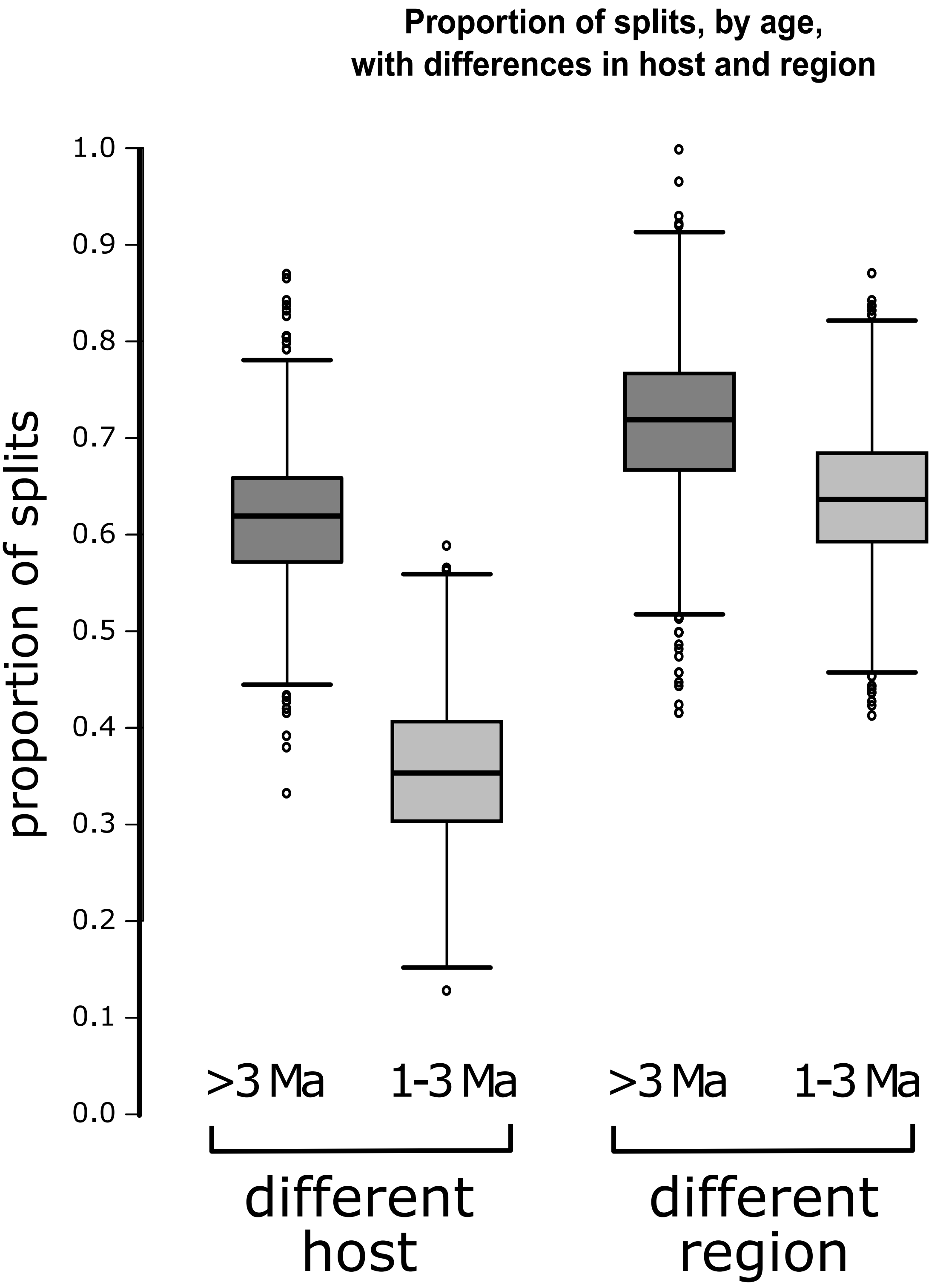

Figure S3. Boxplots summarizing distribution of proportions of lineage splitting events associated with host shifts and dispersal events, by split age, from 2,500 stochastic character mappings simulated on 250 randomly chosen BEAST trees, truncated at 1 Ma. Observed frequencies of each character state among identified specimens were input as Bayesian priors for each simulation.
